# Supplementary material for: Smooth Interpolating Curves with Local Control and Monotone Alternating Curvature
Source: Comput Graph Forum. 2022 Oct 6;41(5):25–38. doi: 10.1111/cgf.14600 (PMC9827861; doi:10.1111/cgf.14600)
Supplement: Supplementary file 1 — Supplement Material [file CGF-41-25-s001.zip › Local-Smooth-Interpolating-MonoCurvature/extern/clothoids/docs/api-matlab/program_listing_file_LineSegment.m.html]

Program Listing for File LineSegment.m — Clothoids v2.0.9

### Navigation

- index
- toc
- Clothoids »
- Program Listing for File LineSegment.m

# Program Listing for File LineSegment.m¶

↰ Return to documentation for file (`LineSegment.m`)

```
classdef LineSegment < CurveBase
  %> MATLAB class wrapper for the underlying C++ class
  methods
    %> Create a new C++ class instance for the Segment object
    %>
    %> **Usage:**
    %>
    %> \rst
    %> .. code-block:: matlab
    %>
    %>    ref = LineSegment(); % (1)
    %>    ref = LineSegment( x0, y0, theta0, L ); % (2)
    %>    ref = LineSegment( x0, y0, theta0, smin, smax ); % (3)
    %>    ref = LineSegment( p0, p1 ); % (4)
    %>
    %> \endrst
    %>
    %> - (1) empty segment
    %> - (2) line segment passing from (x0,y0) at angle theta0
    %> - (3) line segment as in (2) with intial and final curvilinear coordinate respect to (x0,y0)
    %> - (4) segment passing from 2 points
    %>
    %> **On input:**
    %>
    %> - `x0`, `y0`: coordinate of initial point
    %> - `theta0`:   orientation of the circle at initial point
    %> - `L`:        length of curve from initial to final point
    %> - `smin`:     initial curvilinear coordinate of the curve
    %> - `smax`:     final curvilinear coordinate of the curve
    %> - `p0`:       2D point
    %> - `p1`:       2D point
    %>
    %> **On output:**
    %>
    %> - `ref`: reference handle to the object instance
    %>
    function self = LineSegment( varargin )
      self@CurveBase( 'LineSegmentMexWrapper' );
      self.objectHandle = LineSegmentMexWrapper( 'new', varargin{:} );
    end
    % - - - - - - - - - - - - - - - - - - - - - - - - - - - - - - - - - - -
    function str = is_type( ~ )
      str = 'LineSegment';
    end
    % - - - - - - - - - - - - - - - - - - - - - - - - - - - - - - - - - - -
    %>
    %> Buil aline segment suing two points or an origin and a direction
    %>
    %> **Usage:**
    %>
    %> \rst
    %> .. code-block:: matlab
    %>
    %>    ref.build( x0, y0, theta0, L );
    %>    ref.build( p0, p1 );
    %>
    %> \endrst
    %>
    %> **Build 1:**
    %>
    %> - `x0`, `y0` : initial point
    %> - `theta0` : direction of the segment (angle direction)
    %> - `L` : length of the segment
    %>
    %> **Build 2:**
    %>
    %> - `p0` : initial point of the segment
    %> - `p1` : final point of the segment
    %>
    function build( self, varargin )
      LineSegmentMexWrapper( 'build', self.objectHandle, varargin{:} );
    end
    % - - - - - - - - - - - - - - - - - - - - - - - - - - - - - - - - - - -
    %> Return a nurbs representation of the circle segment
    function nurbs = to_nurbs( self )
      nurbs = LineSegmentMexWrapper( 'to_nurbs', self.objectHandle );
    end
    % - - - - - - - - - - - - - - - - - - - - - - - - - - - - - - - - - - -
    %> Return initial and final point of the segment
    function [p1,p2] = points( self )
      [p1,p2] = LineSegmentMexWrapper('points', self.objectHandle );
    end
    % - - - - - - - - - - - - - - - - - - - - - - - - - - - - - - - - - - -
    %> Plot the segment
    %>
    %> **Usage:**
    %>
    %> \rst
    %> .. code-block:: matlab
    %>
    %>   ref.plot();
    %>   ref.plot( 'Color','blue','Linewidth',2);
    %>
    %> \endrst
    %>
    %>
    function plot( self, varargin )
      [ p1, p2 ] = self.points();
      plot( [ p1(1), p2(1) ], [ p1(2), p2(2) ], varargin{:} );
    end
  end
end
```

### Quick search

### Table of Contents

- Matlab Interface Manual
- C++ API
- MATLAB API

«
hide menu

menu
sidebar
»

### Navigation

- index
- toc
- Clothoids »
- Program Listing for File LineSegment.m

© Copyright 2021, Enrico Bertolazzi and Marco Frego.
Created using Sphinx 4.2.0.
